# Thrombospondin-1 promotes fibro-adipogenic stromal expansion and contractile dysfunction of the diaphragm in obesity

**DOI:** 10.1172/jci.insight.175047

**Published:** 2024-07-02

**Authors:** Eric D. Buras, Moon-Sook Woo, Romil Kaul Verma, Sri Harshita Kondisetti, Carol S. Davis, Dennis R. Claflin, Kimber Converso-Baran, Daniel E. Michele, Susan V. Brooks, Tae-Hwa Chun

**Affiliations:** 1 Division of Metabolism, Endocrinology and Diabetes (MEND), Department of Internal Medicine;; 2 Department of Biomedical Engineering;; 3 Department of Surgery, Section of Plastic Surgery;; 4 Department of Molecular and Integrative Physiology; and; 5 Biointerfaces Institute, University of Michigan, Ann Arbor, Michigan, USA.

**Keywords:** Metabolism, Muscle biology, Extracellular matrix, Obesity, Skeletal muscle

## Abstract

Pulmonary disorders affect 40%–80% of individuals with obesity. Respiratory muscle dysfunction is linked to these conditions; however, its pathophysiology remains largely undefined. Mice subjected to diet-induced obesity (DIO) develop diaphragm muscle weakness. Increased intradiaphragmatic adiposity and extracellular matrix (ECM) content correlate with reductions in contractile force. Thrombospondin-1 (THBS1) is an obesity-associated matricellular protein linked with muscular damage in genetic myopathies. THBS1 induces proliferation of fibro-adipogenic progenitors (FAPs) — mesenchymal cells that differentiate into adipocytes and fibroblasts. We hypothesized that THBS1 drives FAP-mediated diaphragm remodeling and contractile dysfunction in DIO. We tested this by comparing the effects of dietary challenge on diaphragms of wild-type (WT) and *Thbs1*-knockout (*Thbs1^–/–^*) mice. Bulk and single-cell transcriptomics demonstrated DIO-induced stromal expansion in WT diaphragms. Diaphragm FAPs displayed upregulation of ECM and TGF-β–related expression signatures and augmentation of a *Thy1*-expressing subpopulation previously linked to type 2 diabetes. Despite similar weight gain, *Thbs1^–/–^* mice were protected from these transcriptomic changes and from obesity-induced increases in diaphragm adiposity and ECM deposition. Unlike WT controls, *Thbs1^–/–^* diaphragms maintained normal contractile force and motion after DIO challenge. THBS1 is therefore a necessary mediator of diaphragm stromal remodeling and contractile dysfunction in overnutrition and a potential therapeutic target in obesity-associated respiratory dysfunction.

## Abstract 2

THBS1 promotes accumulation of Thy1+ fibro-adipogenic progenitors, causing diaphragm muscle fibrosis and dysfunction in obesity

## Graphical abstract

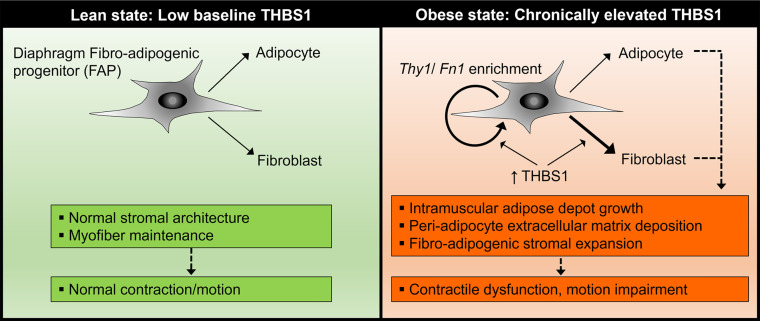

## Introduction

Obesity affects over 40% of Americans (1), predisposing them to respiratory disorders that include dyspnea on exertion (DOE) and obesity hypoventilation syndrome (OHS). DOE affects 30%–80% of people with obesity, while OHS prevalence ranges from 10% in individuals with body mass index (BMI) of 30–35 kg/m^2^ to more than 50% in those with BMI > 50 kg/m^2^ (2–11). DOE reduces the quality of life and impairs exercise tolerance (12–14), while OHS confers 5-year heart failure and mortality rates twice those of demographically matched controls (15). OHS treatment remains medically challenging. Aside from significant weight loss, chronic, noninvasive positive pressure ventilation is its therapeutic mainstay, and permanent tracheostomy is required in severe cases (16).

Clinical studies implicate dysfunction of the respiratory muscles — most notably the diaphragm — as a driver of obesity-associated respiratory impairment (17–20). While its underlying pathophysiology remains unclear, correlations between disordered breathing and increased limb muscle adiposity suggest diaphragm muscle quality may be compromised in people with obesity (21–23). To this end, an autopsy study identified large adipocyte inclusions in the diaphragm of an individual with OHS (24). Intriguingly, patients with obesity have significantly higher mortality rates following COVID-19 infection (25) — a condition shown to promote diaphragm fibrosis (26).

We previously applied a long-term diet-induced obesity (DIO) mouse model to define the relationship between anatomic remodeling and physiologic dysfunction of the diaphragm. In mice subjected to a 6-month high-fat diet (HFD), diaphragm contractile strength declines and inversely correlates with intramuscular adipocyte number and polymerized collagen content (27). In HFD-fed mice, platelet-derived growth factor receptor-α–expressing (PDGFRα-expressing) fibro-adipogenic progenitors (FAPs) are key contributors to intradiaphragmatic accumulation of adipocytes and extracellular matrix (ECM) (27).

FAPs are mesenchymal stem cells that reside within skeletal muscle and give rise to intramuscular adipocytes and ECM-depositing cells (28–30). Important regulators of muscle development and maintenance, FAPs orchestrate muscle stem cell (MuSC) activation and differentiation essential for tissue growth and repair (31–34). Conversely, in mouse models of muscular dystrophy (35, 36) and severe injury (32, 37), disordered FAP dynamics contributes to pathological adiposity and fibrosis associated with contractile dysfunction (38). Recent analyses using single-cell RNA-sequencing (scRNA-Seq) demonstrate distinct FAP populations with pro-remodeling and pro-adipogenic potential (39). Furthermore, a specific FAP subset marked by *THY1* (CD90) expression was associated with fibro-fatty degeneration in quadriceps muscles of individuals with type 2 diabetes (40). Despite these recent advances, molecular mechanisms underlying FAP dysregulation in obesity remain largely unknown.

Thrombospondin-1 (THBS1 or TSP-1) is a matricellular protein present in tissues and circulation. In humans, serum THBS1 levels increase with BMI and are associated with adipose inflammation, insulin resistance, and diabetes (41–44). In mice, *Thbs1* ablation protects against HFD-induced adipose fibrosis while reducing collagen deposition in limb muscles (45). In vitro, THBS1 induces the proliferation of bone marrow–derived mesenchymal cells by activating TGF-β (46). Furthermore, local and circulating THBS1 contribute to fibrotic and degenerative changes in heritable myopathies (47, 48). We previously demonstrated that THBS1 circulates at high levels in mice subjected to DIO (27, 45) and induces the proliferation of diaphragm FAPs (27). Therein, THBS1 is a putative regulator of FAP phenotype and consequent diaphragm remodeling in obesity.

Using the *Thbs1*-null state as an interrogating probe, we aimed to identify FAP subtypes involved in obesity-induced diaphragm remodeling and determine whether *Thbs1* ablation could ameliorate attendant contractile impairment. Our findings indicate that *Thbs1* plays a crucial role in activating TGF-β signaling in diaphragm FAPs and expanding a THY1^+^ FAP subtype. *Thbs1*-knockout (*Thbs1*^–/–^) mice are protected from obesity-induced fibro-adipogenic diaphragm remodeling and respiratory dysfunction.

## Results

### Diaphragm FAP transcriptomic profile and subpopulations change in response to DIO.

**Figure 1:**
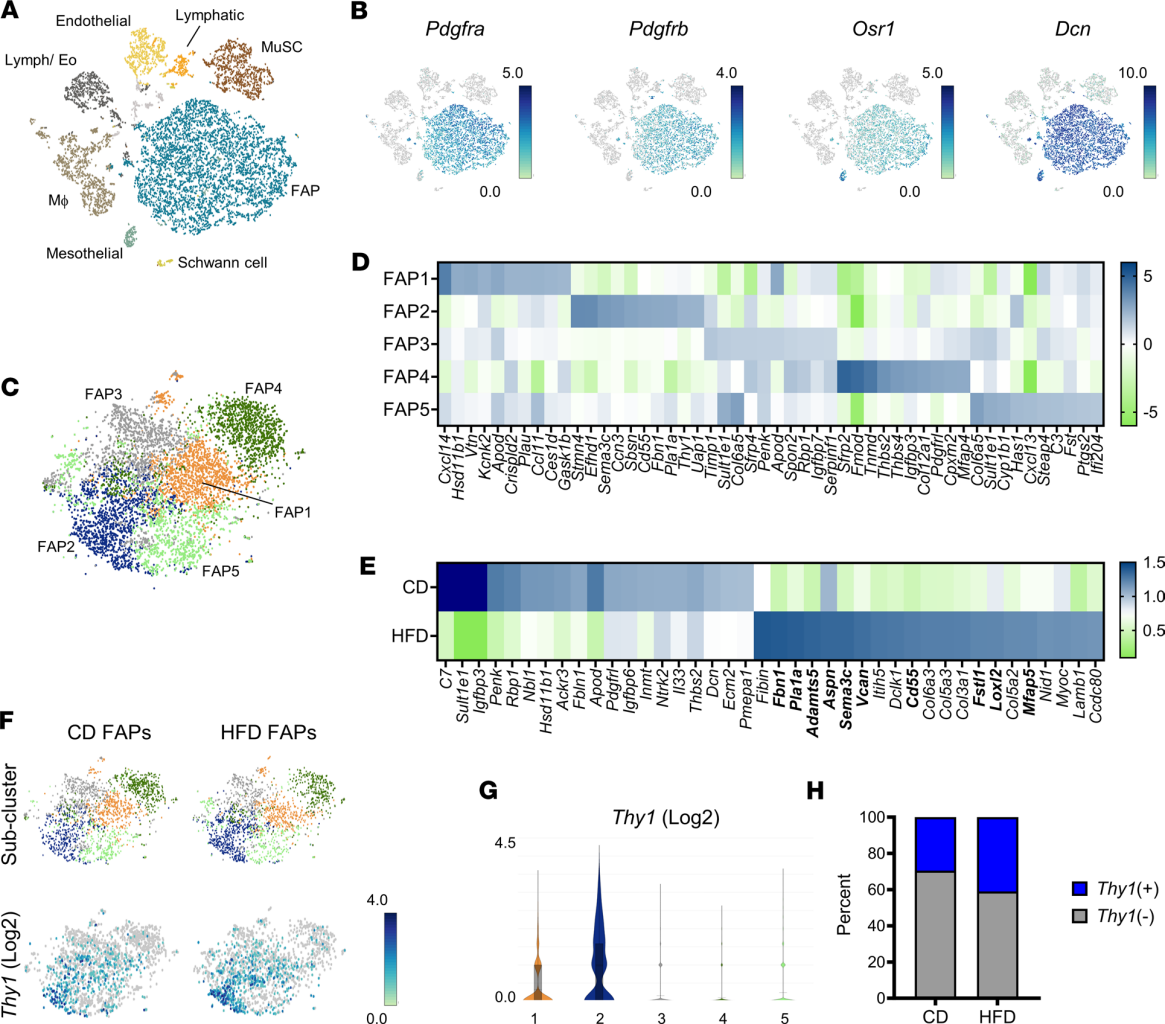
The diaphragm FAP population is heterogeneous and altered by obesity. (**A**) t-Distributed stochastic neighbor embedding (t-SNE) plot: Mononuclear cells from pooled diaphragms of 6-month high-fat diet–fed (HFD-fed) and age-matched control diet–fed (CD-fed) male C57BL/6J mice. *n* = 2 mice per group. (**B**) t-SNE plots: FAP marker genes. (**C**) t-SNE plots: FAP subpopulations from pooled CD and HFD samples. (**D**) Heatmap showing transcripts enriched in FAP subclusters. (**E**) Heatmap showing genes enriched in total FAP populations from CD and HFD samples. Bolded gene names are enriched in the FAP2 subcluster. (**F**) t-SNE and violin plots showing FAP subclusters and *Thy1* expression in CD and HFD samples. (**G**) Violin plots indicating cluster-specific *Thy1* expression. (**H**) Percentage of *Thy1*-expressing among FAPs from CD and HFD samples.

scRNA-Seq has shown FAPs to be a heterogeneous cell type with functionally relevant subpopulations that expand and contract in response to denervation, muscular dystrophy, and injury (34, 49, 50). Transcriptomic changes underlying obesity-associated diaphragm remodeling, on the other hand, remain undefined. To analyze these, we applied scRNA-Seq to mononuclear isolates from costal diaphragms (excluding central tendon and rib attachments) of male C57BL/6J mice subjected to 6-month control diet (CD) (*n* = 2) or high-fat diet (HFD) (*n* = 2) feeding. We computationally aggregated 10x Genomics data to produce a data set comprising 3.4 × 10^8^ reads over 7,906 cells. These resolved into populations of MuSCs (expressing *Myf5*, *Pax7*, and *Cdh15*), endothelial cells (expressing *Flt1*, *Pecam1*, and *Ptprb1*), lymphatic cells (expressing *Clca3a1*, *Ccl21a*, and *Mmrn1*), Schwann cells (expressing *Mpz*, *Ncmap*, and *Kcna1*), and macrophages (expressing *Itgam*, *Cd68*, and *Lyz2*), as well as a heterogeneous leukocyte cluster containing lymphocytes and eosinophils (Figure 1A and Supplemental Figure 1A; supplemental material available online with this article; https://doi.org/10.1172/jci.insight.175047DS1). FAPs, identifiable based on their expression of *Pdgfra*, *Pdgfrb*, *Dcn*, and *Osr1* (29, 51), were the dominant costal diaphragm cell type, accounting for more than 50% of sequenced events (Figure 1, A and B, and Supplemental Figure 1A). A small mesothelial population (with unique enrichment of *Msln*, *Lrn4*, and *Upk3b*) also expressed *Osr1* and *Dcn* but lacked *Pdgfra* and *Pdgfrb* (Figure 1B and Supplemental Figure 1A).

To interrogate their heterogeneity, we reclustered FAPs (4,171 cells) into 5 subpopulations (Figure 1, C and D). The largest of these, FAP1, was enriched in transcripts encoding *G0s2*, *Hsd11b1*, *Vtn*, and *Ccl11* (Figure 1D and Supplemental Figure 1, B and C). This expression signature overlaps with that of the *Cxcl14*-expressing FAP subset previously identified in mouse gastrocnemius and tibialis anterior muscle (52, 53) and closely resembles that of the “adipocyte progenitors” Hepler et al. defined on scRNA-Seq analysis of PDGFRβ^+^ cells from mouse adipose tissue (54). Cells expressing *Mme*, a newly defined marker of adipogenic FAPs (55), were restricted to FAP1 (Supplemental Figure 1C). Few FAPs in any subcluster expressed *Cebpa*, *Pparg*, or *Adipoq* (Supplemental Figure 1D), indicating that the diaphragm FAP pool contains numerous adipocyte progenitors but few committed preadipocytes.

Cells within FAP2 expressed the established FAP marker fibrillin 1 (*Fbn1*), in addition to *Limch*, *Efhd1*, *Smn4*, *Has2*, *Cmah*, and *Dact2* (Figure 1D and Supplemental Figure 2, A–C). The latter signature is consistent with the nonadipogenic, ECM-depositing “fibroinflammatory progenitor” previously described in adipose tissue (54). In agreement, FAP2 cells were enriched in the transcript encoding fibronectin (*Fn1*) (Supplemental Figure 2C). Moreover, FAP2 profile broadly overlapped with that of FBN1^+^ and *Cd55*-expressing FAP populations, respectively identified in mouse and human skeletal muscles (51, 55) (Supplemental Figure 2A). FAP2 cells demonstrated enhanced expression of the genes encoding DPP4 (*Dpp4*) (Supplemental Figure 2B), an adipose stem cell marker (56) that also identifies *Cxcl14*-negative FAPs (52, 53), and endosialin (*Cd248*) (Supplemental Figure 2D), an obesity-associated glycoprotein (57).

Cells within FAP3 highly expressed *Timp1*, a key autocrine regulator of mesenchymal stem cell identity (58). FAP3 cells were further enriched in transcripts expressed within the FAP5 subpopulation, such as that encoding the sulfotransferase SULT1E1 (*Sult1e1*) (Figure 1D and Supplemental Figure 3, A and B). Cells within FAP4 had a more specific signature, expressing transcripts encoding secreted regulators of MuSC differentiation, including the small interstitial leucine-rich proteoglycan fibromodulin (*Fmod*) and soluble Wnt signaling modulator SFRP2 (*Sfrp2*) (59, 60). While FAP4 uniquely contained cells that expressed tenomodulin (*Tnmd*), none within the population expressed the tenocyte marker scleraxis (*Scx*) (61, 62) (Figure 1D and Supplemental Figure 3, C and D).

To assess the impact of dietary modification on the diaphragm FAP population, we analyzed differential gene expression between diaphragm FAPs from CD- and HFD-fed mice. Consistent with our previous work — which showed some diaphragm FAPs assume a pro-fibrotic phenotype in the setting of DIO (27) — FAPs from HFD samples exhibited enhanced expression of genes encoding ECM species, such as *Col3a1*, *Col5a2*, *Col5a3*, and *Col6a3* (Figure 1E). In addition, HFD FAPs were enriched in FAP2 transcripts: *Fbn1*, *Pla1a1*, *Sema3c*, *Cd55*, *Mfap5*, and *Thy1* (Figure 1E and Supplemental Figure 2, A and C). The latter was particularly intriguing, given a recent report describing an increase in THY1^+^ FAPs in obese individuals with type 2 diabetes, who demonstrate fibro-fatty muscle degeneration (40). In our data set, *Thy1* transcript was largely restricted to FAP2 (Figure 1, F and G), while *Thy1*-expressing cells likewise expressed FAP2 marker genes (Supplemental Figure 4, A and B). Notably, *Thy1*-expressing FAPs were more numerous in samples from mice fed HFD than CD (Figure 1H).

Taken together, these data demonstrate that diaphragm FAPs resolve into distinct subpopulations, suggesting compartmentalization of adipogenic, ECM deposition, and regulatory functions. HFD promotes a pro-fibrotic transcriptomic signature and FAP2 subpopulation enrichment, with a corresponding increase in *Thy1*-expressing cells. These findings corroborate our earlier demonstration of increased fibrogenic FAPs within the obese diaphragm (27) while revealing similarities between the diaphragms of DIO mice and limb muscles of humans with obesity and type 2 diabetes (40).

### THBS1 underlies quantitative and qualitative changes in diaphragm FAPs during the DIO challenge.

**Figure 2:**
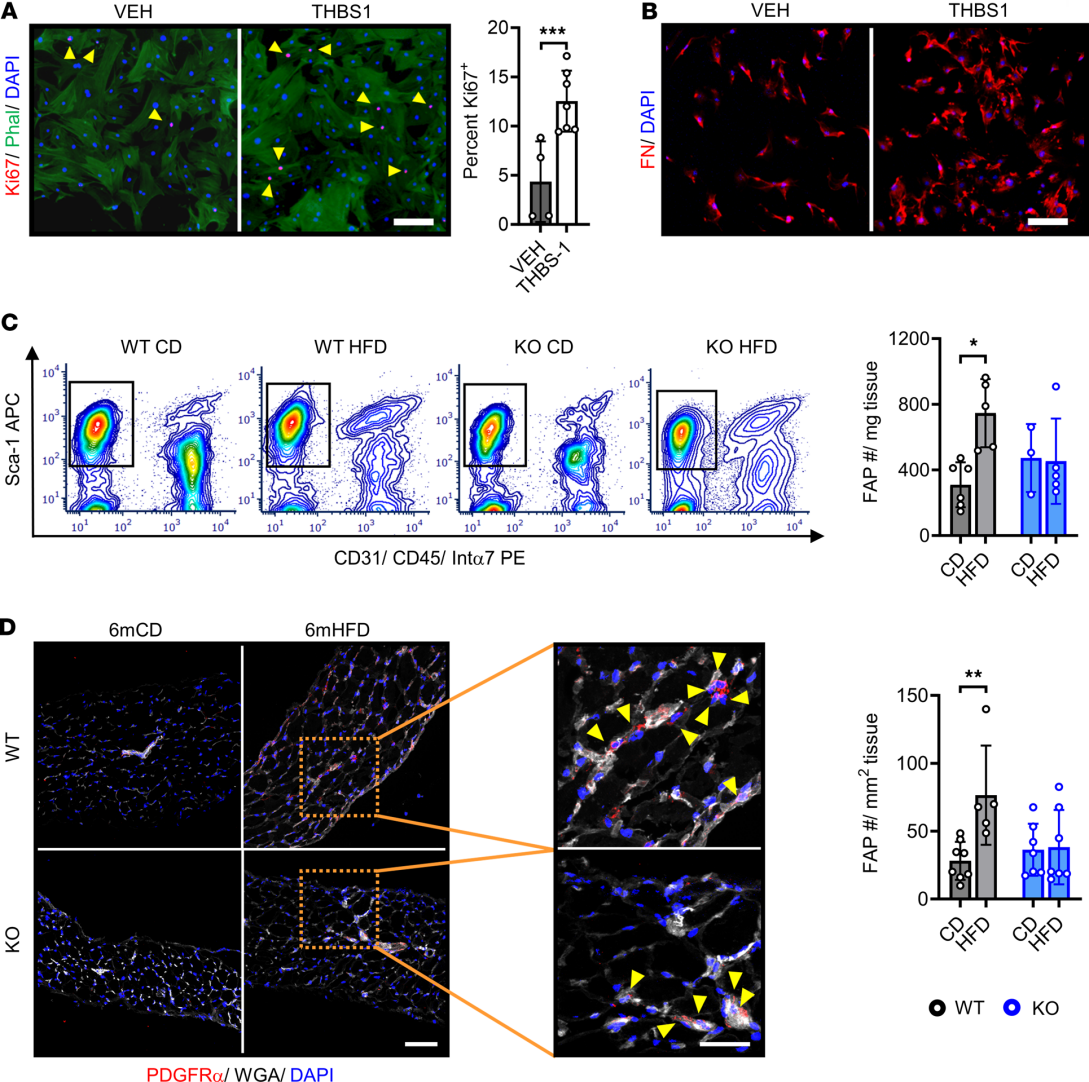
High-fat diet feeding causes THBS1-dependent FAP population expansion. (**A**) Primary FACS-isolated FAPs treated with THBS1 (5 μg/mL) or DMEM vehicle (VEH) and subjected to Ki67 immunocytochemistry with phalloidin (PHAL) counterstain. Scale bar: 50 μm. Arrowheads indicate Ki67^+^ nuclei. The bar graph indicates percentage Ki67^+^ cells. *n* = 2 unique experiments per group with 4–7 replicates per experiment. (**B**) Primary FAPs treated as indicated in **A** and subjected to fibronectin (FN) immunocytochemistry. Representative images from 2 unique experiments with 3–4 replicates per experiment. Scale bar: 50 μm. (**C**) Analysis of FAPs from costal diaphragm tissue of wild-type (WT) and *Thbs1^–/–^* (KO) mice fed a control diet (CD) or high-fat diet (HFD) for 6 months. Left panels show representative flow cytometry plots. FAPs are positive for Sca-1 and negative for CD31, CD45, and integrin α_7_ (Intα7). Right panel shows bar graph quantifying FAPs/mg tissue. Each sample contains 2 whole costal diaphragms. *n* = 3–6 samples (6–12 mice) per group. (**D**) Immunohistochemistry for PDGFRα, with wheat germ agglutinin (WGA) counterstain, in diaphragm samples from WT and KO mice fed CD or HFD for 6 months. Scale bar: 100 μm in main panel, 50 μm in inset. Arrowheads in inset indicate FAPs, defined as PDGFRα staining surrounding a DAPI^+^ nucleus. Bar graph indicates the quantification of PDGFRα^+^ cells/mm^2^ tissue cross-sectional area (CSA). *n* = 5–8 mice per group. Statistical analysis with *t* test for individual comparisons, 2-way ANOVA for multiple variable comparisons. Error bars indicate mean ± SD. *P < 0.05, **P < 0.01, ***P < 0.001.

We next sought to identify molecular regulators of DIO-induced FAP profile changes and focused on the obesity-associated growth factor THBS1 (27, 44–46). To ascertain its impact on FAP biology in vitro, we isolated FAPs with fluorescence-activated cell sorting (FACS) using an established surface marker profile (Sca-1^+^, CD31^–^, CD45^–^, integrin α_7_^–^) (28, 29), then applied THBS1 at a concentration observed in the plasma of humans with obesity (44). THBS1 administration induced FAP proliferation, as indicated by increased number of Ki67-labeled cells (Figure 2A). Furthermore, THBS1-treated cells demonstrated enhanced extracellular deposition of FAP2-associated fibronectin (Figure 2B).

Given the high level of circulating THBS1 in HFD-fed mice (27), we hypothesized that THBS1 contributed to DIO-associated FAP changes in the diaphragm. To test this, we subjected whole-body *Thbs1*^–/–^ (KO) mice (45) (Supplemental Figure 5, A and B) and wild-type (WT) controls to 6-month HFD-feeding, then evaluated diaphragm FAPs by flow cytometry, immunohistochemistry (IHC), and scRNA-Seq.

As previously reported (43), KO mice displayed slightly reduced linear body growth during adulthood versus WT animals (Supplemental Figure 5C). Nonetheless, they gained weight with HFD feeding (Supplemental Figure 5, D and E) such that their body composition was equivalent to that of HFD-fed WT mice (Supplemental Figure 5F). Additionally, both WT and KO mice fed HFD showed similarly impaired glucose tolerance compared with age-matched WT mice fed CD (Supplemental Figure 5G). WT and KO animals also demonstrated comparable DIO-induced increases in liver, perigonadal adipose, and inguinal adipose weights (Supplemental Figure 6, A–C). Finally, weights of several limb muscles did not significantly differ between groups regardless of diet (Supplemental Figure 6, D–H).

**Figure 3:**
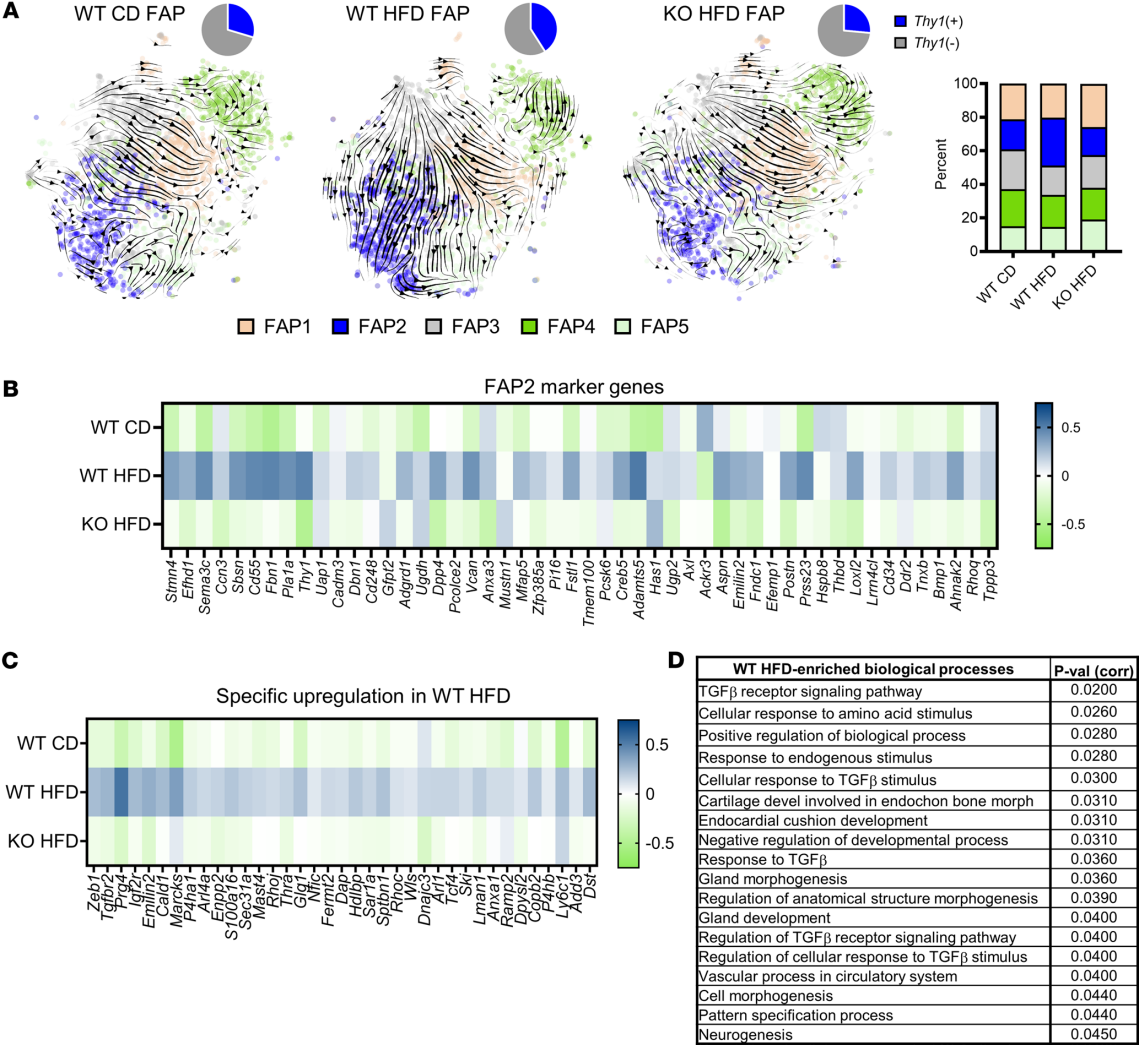
*Thbs1* is required for obesity-induced FAP subpopulation shifts. (**A**) Velocity plots demonstrating temporal relationships between FAP subpopulations in wild-type (WT) mice fed control diet (CD) or high-fat diet (HFD) for 6 months and *Thbs1^–/–^* (KO) mice fed HFD for 6 months. Arrow directions represent the trajectory of differentiation between subpopulations. Thickness of arrow indicates a rate of change. Pie charts indicate percentage of *Thy1*-expressing cells in each group. Stacked bar graph shows proportion of individual FAP subpopulations in each group. *n* = 2 mice per group. (**B**) Heatmap indicating the expression of FAP2 marker genes in each group. (**C**) Heatmap showing genes specifically enriched in the WT HFD group. (**D**) Gene Ontology terms (Biological Processes) specifically enriched in WT HFD FAPs, with corrected *P* values.

Using flow cytometry, we compared mononuclear isolates (2 whole costal diaphragms, excluding central tendon and rib attachment, per sample) from WT and KO mice fed either CD or HFD for 6 months (*n* = 3–6 samples per group), then quantified FAPs/mg tissue. In WT mice, FAP quantity increased with HFD feeding. On the contrary, KO mice had an equivalent FAP/mg tissue, regardless of dietary condition (Figure 2C). We corroborated these findings by performing IHC for FAP marker PDGFRα on frozen costal diaphragm samples (*n* = 5–8 animals per group), observing HFD-induced FAP number increase within WT but not KO mice (Figure 2D). We then asked whether KO mice were also protected from DIO-induced changes in FAP transcriptomics. To assess this, we integrated samples from 6-month HFD-fed KO animals into our scRNA-Seq framework. In diaphragm mononuclear isolates from WT CD (*n* = 2), WT HFD (*n* = 2), and KO HFD (*n* = 2) mice (5.2 × 10^8^ reads over 12,275 cells), we focused our attention on FAPs (6,591 cells). Velocity analysis of these data demonstrated DIO induced differentiation from *Timp1*-expressing FAP3 progenitors toward FAP2. In WT mice, this produced a quantitative increase in the FAP2 population and a commensurate increase in *Thy1*-expressing cells (Figure 3A). In contrast, HFD-fed KO mice were protected from these population shifts and maintained FAP2 and *Thy1*-expressing cell content like that of WT mice fed CD (Figure 3A). In agreement, total FAP samples from WT HFD mice showed selective enrichment of marker genes for FAP2 (Figure 3B) and *Thy1*-expressing cells (Supplemental Figure 7A).

We next used iPathwayGuide to examine genes and biological pathways enriched in each group (Supplemental Figure 7B). Several ECM-related genes — *Adamts5*, *Sema3c*, *Itih5*, *Cd55*, *Fbn1*, *Col3a1*, *Col4a1*, *Col4a2*, and *Col6a3* — were upregulated in both HFD-fed WT and KO FAPs compared with those of WT mice fed CD; however, their expression levels were highest in WT HFD samples (Supplemental Figure 7C). Genes with specific upregulation in the WT HFD group included those encoding proteins involved in TGF-β signaling and myofibroblastic transition (e.g., *Zeb1*, *Tgfbr2*, and *Prg4*) (63–65) (Figure 3C and Supplemental Figure 7, D and E). In agreement, numerous TGF-β–associated pathways were enriched in WT HFD (Figure 3D and Supplemental Figure 8A) but not in WT CD or KO HFD samples (Supplemental Figure 8, A–E). Given these findings, we sought to determine whether the mitogenic impact of THBS1 on FAPs (Figure 2, A and B) depended on TGF-β. Indeed, we observed that cotreatment with SB-431542, an inhibitor of TGF-β signaling (66), ameliorated THBS1-induced FAP proliferation, while dramatically inhibiting fibronectin deposition (Supplemental Figure 9, A–C).

We then analyzed TGF-β receptor expression within FAP subtypes and found *Tgfbr2* to be enriched in FAP2. On the contrary, other transcripts encoding receptors for TGF-β and PDGF species (i.e., *Tgfbr1*, *Tgfbr3*, *Pdgfra*, and *Pdgfrb*) were expressed equally across FAP populations (Supplemental Figure 10, A and B). Interestingly, the gene encoding CD47 — a cell surface THBS1 receptor that acts as a “don’t eat me signal” to inhibit macrophage-mediated phagocytosis of fibroblasts and other cell types (67) — was also enriched in FAP2 (Supplemental Figure 10C). Conversely, the gene encoding the established endothelial THBS1 receptor, CD36 (68), was negligibly expressed in FAPs of all subtypes (Supplemental Figure 10C).

Together, these data demonstrate that THBS1 is required for DIO-induced expansion of FAP pool and its shift toward *Thy1*-expressing FAP2 cells. Increased TGF-β signaling — known to be activated by THBS1 (46) and elevated in DIO (69) — likely underlies key aspects of this phenotypic switch.

### Whole tissue transcriptomics highlights reduced stromal gene expression in obese Thbs1^–/–^ mice.

**Figure 4:**
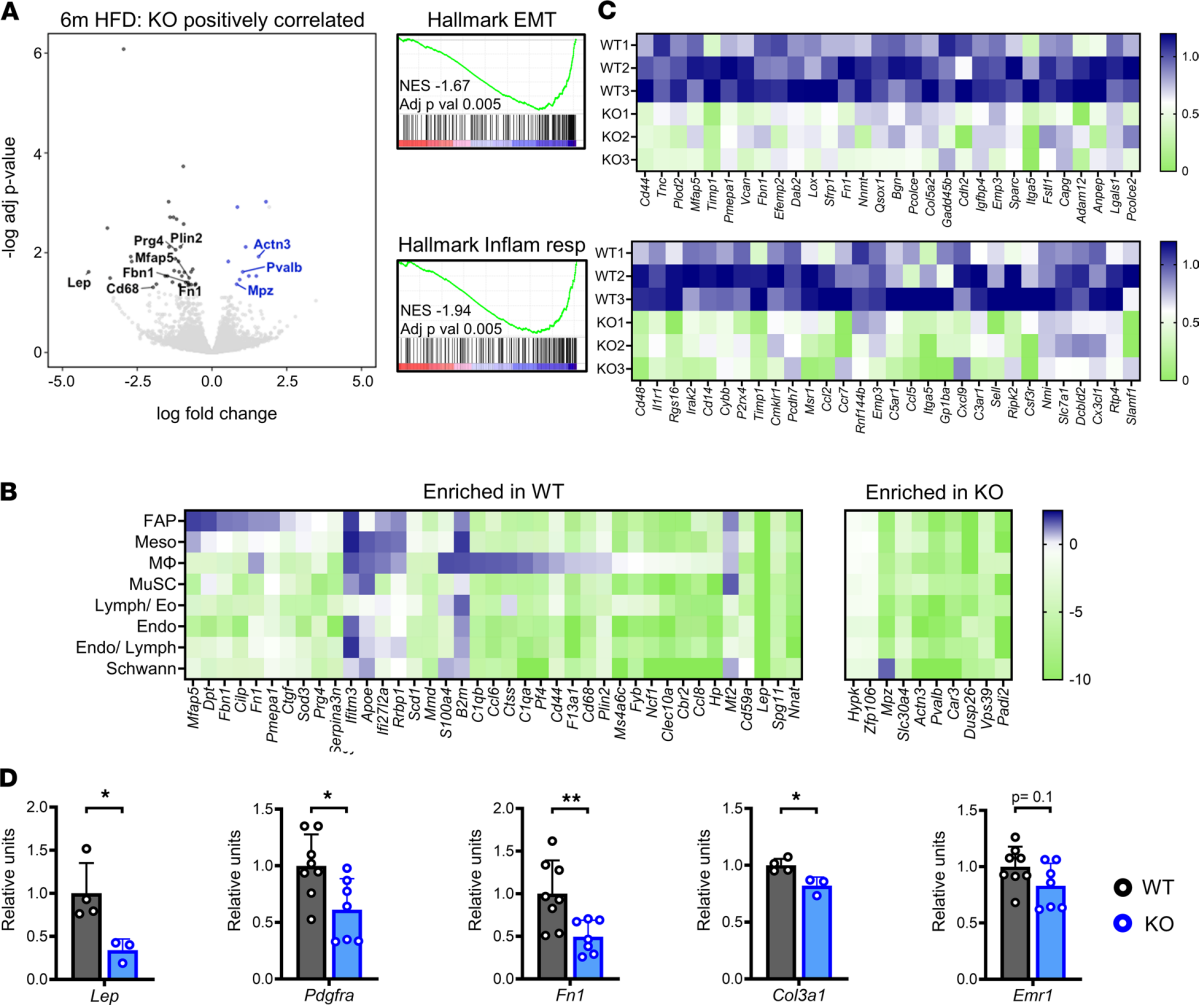
Whole tissue transcriptomics highlights enrichment of stromal genes in wild-type versus *Thbs1^–/–^* mice subjected to DIO. (**A**) Volcano plot of whole costal diaphragm RNA-Seq demonstrating differentially expressed genes between wild-type (WT) and *Thbs1^–/–^* (KO) mice fed high-fat diet (HFD) for 6 months (6m HFD). *n* = 3 mice per group. *X* axis indicates log fold-change (FC) in KO versus WT. *Y* axis indicates –log adjusted *P* value. (**B**) Heatmap integrating tissue-level RNA-Seq with scRNA-Seq data. Genes indicated are those enriched in WT and KO mice on bulk RNA-Seq (i.e., the points on the volcano plot in **A**). Cell types are those identified on scRNA-Seq (as shown in Figure 1A). Heatmaps show cell type–specific expression as defined on scRNA-Seq. (**C**) Enrichment plots demonstrating selected HALLMARK pathways differentially expressed between 6m HFD-fed WT and KO mice: epithelial mesenchymal transition (Hallmark EMT) and inflammatory response (Hallmark Inflamm resp). Heatmaps show the expression of leading-edge genes in individual samples. (**D**) qPCR analysis of selected genes performed on costal diaphragm tissue of 6m HFD WT and 6m HFD KO mice. *n* = 3–8 whole hemidiaphragm samples per group. Statistical analysis with *t* test. Error bars indicate mean ± SD. *P < 0.05, **P < 0.01.

To determine whether FAP profile differences between HFD-fed WT and KO mice corresponded with gene expression changes at the tissue level, we performed bulk RNA-Seq on whole costal diaphragm samples (*n* = 3 diaphragms per group) from these animals. This analysis revealed a pronounced difference in the transcript encoding adipocyte marker leptin (*Lep*), which was expressed more in WT samples (Figure 4A). We performed an integrated analysis to determine whether other differentially expressed transcripts identified by bulk RNA-Seq were enriched in any of the specific mononuclear cell types defined by scRNA-Seq. This approach showed that numerous genes more highly expressed in HFD-fed WT diaphragm tissue were specifically enriched in FAPs (*Mfap5*, *Dpt*, *Fbn1*, *Cilp*, *Fn1*, *Pmepa1*, *Ctgf*, *Sod3*, and *Prg4*) and macrophages (*S100a4*, *C1qb*, *Ccl6*, *Ctss*, *C1qa*, *Pf4*, *Cd44*, *F13a1*, *Cd68*, and *Plin2*) (Figure 4, A and B). On the contrary (aside from Schwann cell marker *Mpz*), transcripts enriched in HFD-fed KO samples were not enriched in any mononuclear cell type. In fact, genes relatively overexpressed in the KO diaphragm included well-known myofiber transcripts, like those encoding parvalbumin (*Pvalb*) and α-actinin-3 (*Actn3*) (Figure 4, A and B). Consistent with this, gene set enrichment analysis (GSEA) demonstrated the HALLMARK epithelial mesenchymal transition pathway (which contains ECM-related genes) and inflammatory response pathway (which contains macrophage-related genes) to be enriched more in WT than KO samples (Figure 4C). Quantitative PCR (qPCR) analysis substantiated these findings, showing *Thbs1* ablation to reduce levels of *Lep*, *Pdgfra*, *Fn1*, and *Col3a1* in diaphragms of HFD-fed mice (Figure 4D).

Expression of *Emr1*, encoding macrophage marker F4/80, also trended down in KO samples; however, the difference did not reach statistical significance (Figure 4D). CD68 IHC on costal diaphragm sections further demonstrated HFD feeding to raise tissue macrophage number in WT mice. This increase was less pronounced in KO animals (Supplemental Figure 11A); however, total macrophage number, even in WT HFD-fed mice, was 2- to 3-fold lower than that of FAPs (Figure 2C). Furthermore, analysis of scRNA-Seq data showed lipid-associated *Trem2*-expressing macrophages (70) to exist in equal proportion in samples from HFD-fed WT and KO mice (Supplemental Figure 11B).

Taken together, these data show *Thbs1* ablation reduces expression of stromal genes, particularly those specific to adipocytes, FAPs, and macrophages, in the obese diaphragm. Of the latter 2 cell types, FAPs are likely greater contributors to tissue-level phenotype, given their higher numbers. The relative increase in muscle-specific transcripts within the KO diaphragm suggests an increased muscle/stroma ratio in the setting of *Thbs1* ablation.

### Thbs1 ablation protects against diaphragm fibro-adipogenic remodeling.

We previously demonstrated that DIO promotes diaphragm tissue remodeling, characterized by increased FAP-derived ECM-depositing cells and intramuscular adipocytes (27). Given that *Thbs1* ablation ameliorated FAP population expansion and subtype shifts while reducing adipocyte and ECM-related transcripts, we surmised that KO mice would be protected from the remodeling phenotype.

**Figure 5:**
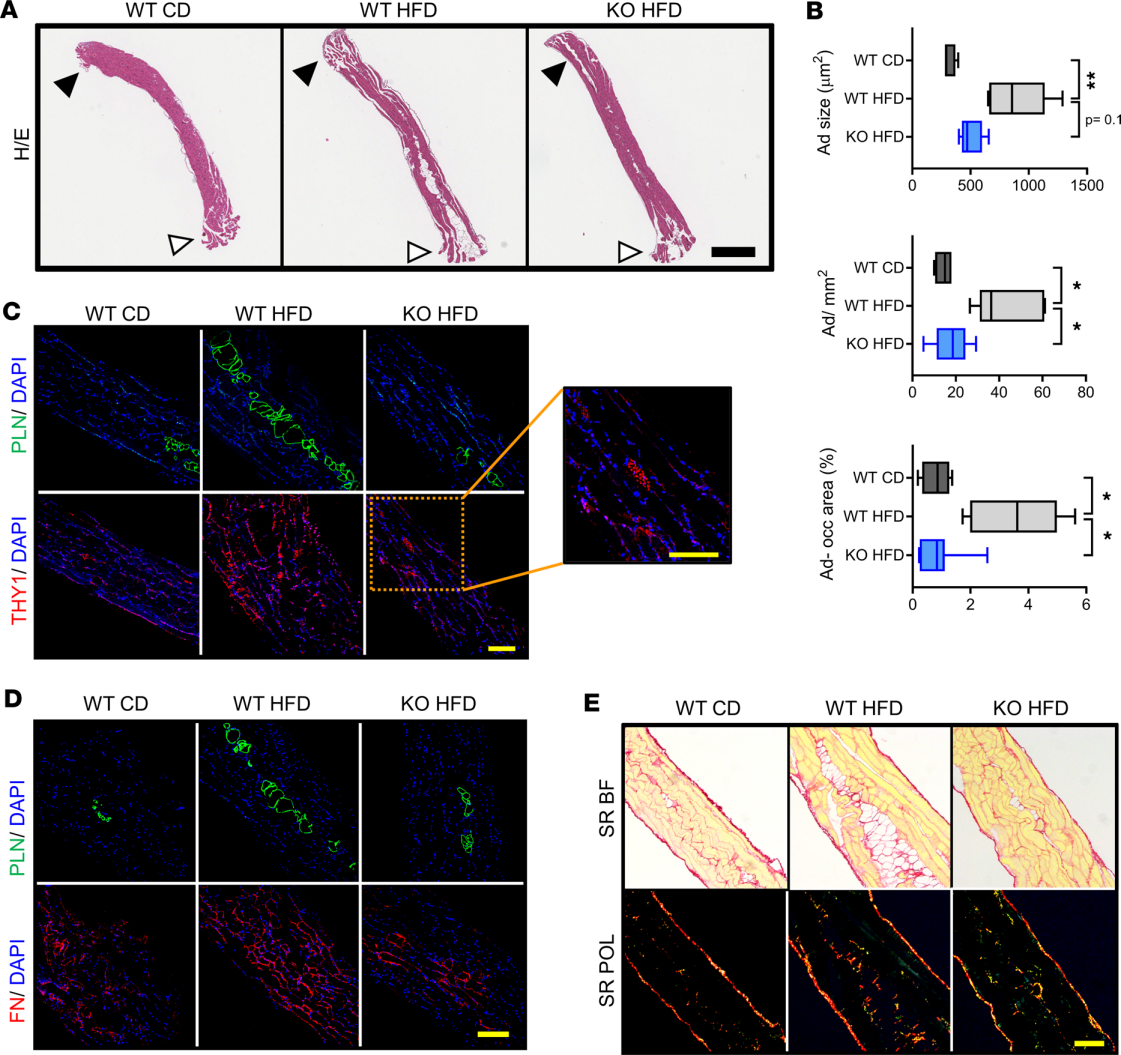
*Thbs1* ablation protects against diaphragm fibro-adipogenic remodeling. (**A**) H&E-stained longitudinal diaphragm sections from wild-type mice fed control diet (WT CD) or HFD (WT HFD) for 6 months and *Thbs1^–/–^* mice fed HFD for 6 months (KO HFD). White arrowhead indicates rib attachment point. Black arrowhead indicates central tendon attachment point. Scale bar: 600 μm. Representative samples from 5–7 mice per group. (**B**) Adipocyte size, adipocyte number/millimeter cross-sectional area (CSA), and percentage total CSA occupied by adipocytes in samples described in **A**. Values are the average of measurements made on 3 nonconsecutive 7 μm–thick sections encompassing the entire rib-to-tendon extent of muscle. *n* = 4–7 mice per group. Box indicates 25th–75th percentile, midline indicates median, and whiskers indicate minimum and maximum values. (**C**) Immunofluorescence staining of perilipin (PLN) and THY1 on adjacent 7 μm–thick longitudinal sections from animals described above. Representative images from analysis of 5–7 mice per group. Inset indicates THY1 staining of a nerve passing through the sample, representing an internal positive-staining control. Scale bar: 200 μm. (**D**) PLN and fibronectin (FN) staining on adjacent 7 μm–thick longitudinal sections from animals described above. Representative images from analysis of 5–7 mice per group. Scale bar: 200 μm. (**E**) Picrosirius red (SR) staining of 7 μm–thick longitudinal sections from animals described above. Bright-field (BF) and polarized light (POL) images: polymerized collagens fluoresce red/yellow under POL. Representative images from analysis of 5–7 mice per group. Scale bar: 200 μm. Statistical analysis with Kruskal-Wallis test for nonparametric multiple comparisons. *P < 0.05, **P < 0.01.

To assess this, we examined tissue morphology in H&E-stained longitudinal costal diaphragm sections spanning the rib and tendon attachment points (*n* = 5–7 mice per group, 3–4 nonconsecutive sections per animal) (Figure 5A). In WT mice, this analysis demonstrated intramuscular adipocyte inclusions — their identity confirmed by staining for the lipid droplet protein perilipin (Supplemental Figure 12A) — that were particularly prominent in the lateral costal diaphragm and larger in mice subjected to 6-month DIO (Figure 5A). Indeed, intramuscular adipocyte size and number, as well as tissue cross-sectional area (CSA) occupied by adipocytes, increased in HFD- versus CD-fed animals (Figure 5B and Supplemental Figure 12A). Despite similar weight gain to WT mice, KO mice were largely protected from obesity-associated intramuscular adiposity (Figure 5, A and B, and Supplemental Figure 12A).

We next sought to understand the geographical relationship between these intramuscular adipose depots and THY1^+^ FAPs. Immunofluorescence staining of adjacent sections for perilipin and THY1 demonstrated collections of THY1^+^ cells close to adipocyte inclusions; their number was higher in HFD-fed WT samples versus the other groups (Figure 5C). Moreover, prominent deposition of both fibronectin (Figure 5D) and polymerized collagen (Figure 5E) surrounded adipose depots. Both increased with HFD feeding in WT mice, while samples from HFD-fed KO mice resembled those of WT mice fed CD. Consistent with the higher expression of *Col3a1* in HFD-fed WT versus KO tissue, intradiaphragmatic collagen 3 deposition also increased with DIO in a *Thbs1*-dependent manner (Supplemental Figure 12B).

We next examined a diaphragm autopsy sample from a 62-year-old individual with obesity (BMI 32 kg/m^2^) and observed clusters of intramuscular adipocytes akin to those seen in diaphragms of WT HFD-fed mice (Supplemental Figure 12C). These intramuscular depots were larger than those seen in a sample from a 72-year-old individual of healthy BMI (22 kg/m^2^), and like in the DIO mouse model, associated with areas of increased fibronectin deposition and THY1^+^ cells (Supplemental Figure 12D).

Notably, diaphragm samples from HFD-fed WT and KO mice did not significantly differ in thickness (Supplemental Figure 13A), myofiber CSA (Supplemental Figure 13B), or myofiber type (Supplemental Figure 13C). Together, these histological analyses provide evidence that *Thbs1* ablation protects the diaphragm from DIO-induced fibro-adipogenic remodeling in a manner consistent with the effects predicted from transcriptomic profiles at the cell and tissue level. Moreover, the findings indicate that DIO-induced, *Thbs1*-dependent increases of intramuscular adiposity are geographically coupled with ECM deposition and abundant THY1^+^ cells in a pattern that resembles the histology of the obese human diaphragm.

### The Thbs1^–/–^ diaphragm preserves its contractile force in the setting of DIO challenge.

**Figure 6:**
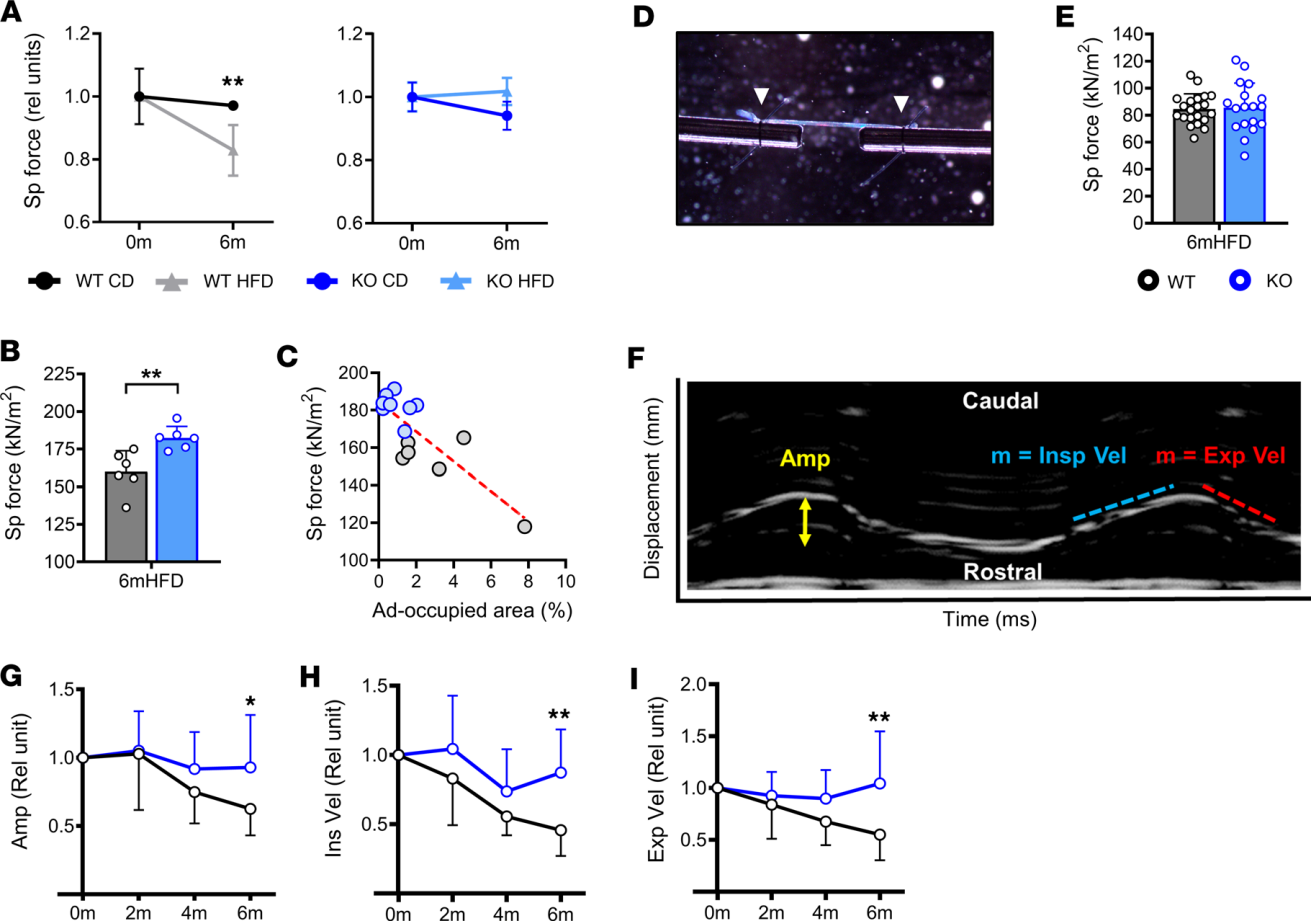
DIO challenge compromises diaphragm force and motion in wild-type but not *Thbs1^–/–^* mice. (**A**) Isometric specific force (Sp force) of wild-type (WT) and *Thbs1^–/–^* (KO) mice (normalized to baseline, relative units) at baseline (0m) and following 6-month (6m) control diet (CD) or high-fat diet (HFD) feeding. *n* = 4–6 animals per group; 1–2 diaphragm strips per animal averaged. (**B**) Isometric specific force (absolute value) of samples from 6m HFD WT and KO mice. *n* = 6 animals per group; 1–2 diaphragm strips per animal averaged. (**C**) Correlation plot demonstrating the relationship between isometric specific force and percentage tissue cross-sectional area occupied by adipocytes in diaphragm strips subjected to isometric force testing. 6m HFD WT and KO mice, 8–9 individual muscle strips per group. (**D**) Image of single myofiber undergoing isometric force testing. White arrowheads indicate sutures affixing fiber to force transducer-servomotor apparatus. (**E**) Isometric specific force of single myofibers isolated from 6m HFD WT and KO mice (*n* = 4–5 animals per group; 4–5 fibers per animal). (**F**) Diaphragm ultrasound M-mode tracing with measured parameters labeled. *X* axis represents time; *y* axis represents displacement along the rostral-caudal axis. (**G**–**I**) Diaphragm motion parameters: amplitude (Amp), inspiratory velocity (Ins Vel), expiratory velocity (Exp Vel), normalized to baseline measured at 0, 2, 4, and 6 m. *n* = 8–9 animals per group. Statistical analysis with *t* test for individual comparisons and linear regression for correlational analysis. Error bars indicate mean ± SD. *P < 0.05, **P < 0.01.

Given the improved tissue architecture observed in HFD-fed KO versus WT mice, we predicted that *Thbs1* ablation would also protect the diaphragm from obesity-associated mechanical dysfunction. To test this hypothesis, we performed ex vivo isometric force testing on diaphragm strips (Supplemental Figure 13D) isolated from WT and KO mice at baseline (2 months old) and following 6-month CD or HFD feeding (*n* = 4–6 mice per group, 1–2 diaphragm samples per mouse). In WT animals, 6-month CD effected no difference in isometric force versus baseline. HFD, on the other hand, caused specific force to decline by nearly 20% (Figure 6A). In KO mice, specific force values remained unchanged from baseline regardless of whether animals received CD or HFD (Figure 6A). In addition, samples from 6-month HFD-fed KO mice had significantly higher specific force measurements than those of HFD-fed WT mice (Figure 6B). Therefore, *Thbs1* ablation protects the diaphragm from obesity-associated contractile force reduction.

Within diaphragm muscle strips from HFD-fed WT and KO mice subjected to isometric testing, we observed a negative correlation between adipocyte-occupied CSA and measured specific force (Figure 6C). Given this relationship, we surmised that tissue-level contractile force deficits resulted from altered muscle architecture. An alternative explanation is that THBS1 could directly impair myofiber function in obesity. To test this possibility, we performed isometric force testing on single myofibers isolated from WT and KO mice fed HFD for 6 months (Figure 6D). This analysis found specific force measurements of individual fibers from mice of each group to be statistically indistinguishable (Figure 6E and Supplemental Figure 13E). As such, the protective effect of *Thbs1* ablation is not dependent on better sarcomere contractility but instead may result from undisrupted tissue architecture necessary for coordinated muscle contraction.

### Thbs1 ablation protects mice from obesity-associated deterioration of diaphragm motion.

We then asked whether the preservation of normal diaphragm contraction seen in HFD-fed KO mice translated into protection from obesity-associated respiratory dysfunction. To assess this, we subjected WT and KO mice to a 6-month HFD time course and serially analyzed diaphragm motion with noninvasive ultrasound. M-mode measurements, plotted with time on the *x* axis and diaphragm displacement on the *y* axis, enabled measurement of diaphragm excursion amplitude, inspiratory velocity, and expiratory velocity (Figure 6F) (27, 71). In WT mice, these parameters progressively declined with HFD feeding duration. On the contrary, in KO mice, all measurements remained stable throughout the time course (Figure 6, G–I). Moreover, at the 6-month time point, baseline-normalized amplitude, inspiratory velocity, and expiratory velocity were significantly higher in KO than WT mice (Figure 6, G–I). In sum, animals lacking *Thbs1* are protected from obesity-associated diaphragm motion compromise.

## Discussion

Our findings demonstrate that anatomic remodeling and contractile dysfunction of the diaphragm are interrelated, *Thbs1*-dependent obesity complications. In the setting of long-term overnutrition, THBS1 promotes stromal expansion characterized by increased THY1^+^ FAPs, aberrant ECM deposition, and elevated intramuscular adiposity. These changes correspond with a decline in tissue-level isometric force generation — independent of single myofiber sarcomere function — contributing to reduced diaphragm motion.

Our results define THBS1 as a key regulator of quantitative expansion and qualitative changes in the diaphragm FAP pool during long-term DIO. A circulating matricellular protein, THBS1 is produced by megakaryocytes, platelets, leukocytes, endothelial cells, fibroblasts, and adipocytes (41, 72–74). In most cell types, THBS1 expression is low at baseline but acutely rises in wound healing and ischemic stress responses (75). Persistent, maladaptive THBS1 elevation occurs with aging and prolonged nutritional stress. For instance, *THBS1* expression increases in adipose depots of obese humans and positively correlates with the degree of insulin resistance (41), while plasma THBS1 concentrations are higher in patients with impaired glucose tolerance (44, 76). Rodent metabolic syndrome models recapitulate these findings (45), as evidenced by increased circulating THBS1 levels observed in DIO mice (27, 45).

Deposited in the ECM, THBS1 induces context-specific trophic effects, both proliferation and ECM production, on stromal cells (75). In cultured bone marrow–derived mesenchymal cells, THBS1 acts as a potent mitogen (46). Our in vitro data demonstrate an analogous effect on FAPs, as THBS1 concentrations comparable to those found in humans with obesity (44) promote their proliferation. Analysis of FAPs from diaphragms of KO mice supports the relevance of this effect in vivo: unlike WT mice, which undergo expansion of the FAP pool with obesity, KO mice maintain similar FAP numbers to baseline when challenged with DIO. Mechanistically, THBS1 induces established FAP mitogens, specifically facilitating the conversion of latent to active TGF-β (46). Our transcriptomic analyses and in vitro experiments highlight TGF-β as a likely driver of *Thbs1*-dependent FAP proliferation. These data further implicate THBS1 in the augmented TGF-β signaling previously described in humans with metabolic syndrome (77). Of note, parallel impacts of THBS1 on other trophic factors or FAP survival (32, 40, 78) — for instance, via reduced macrophage-mediated clearance (67) — might also regulate diaphragm FAP pool size.

THBS1 promotes mesenchymal ECM production in numerous tissues (79), and this process appears operative in diaphragm FAPs. In vitro, THBS1 induces deposition of fibronectin, an ECM molecule transcriptionally regulated by TGF-β (80). In vivo, FAPs isolated from the obese diaphragm assume a fibrogenic signature. THBS1 is required for this shift, since the expression of numerous obesity-induced, ECM-related FAP genes (such as *Fbn1*, *Col3a1*, and *Adamts5*) is markedly blunted in HFD-fed KO mice. Moreover, some of the genes most upregulated in WT HFD versus WT CD and KO HFD FAPs include species involved in TGF-β signaling (e.g., *Zeb1*, *Tgfbr2*, and *Prg4*).

scRNA-Seq–based subclustering defined several diaphragm FAP subpopulations, some exhibiting considerable transcriptomic overlap with those described previously. The most notable was *Thy1*-expressing FAP2 — a cell type similar to the fibro-inflammatory progenitor described in visceral and subcutaneous adipose tissue and the FBN1^+^ and *Cd55*-expressing FAP subsets identified in human and mouse skeletal muscle (51, 54, 55). In our model, the FAP2 population arose from *Timp1*-expressing FAP3 precursors and expanded with DIO, shifting the total FAP pool toward enrichment of FAP2 markers. We noted a striking resemblance between FAP2 and the THY1^+^ FAPs previously shown to increase in individuals with type 2 diabetes. In these human samples, the presence of THY1^+^ cells was associated with fibro-fatty muscle remodeling (40) like that seen in DIO mice (27) and our diaphragm autopsy samples. Furthermore, THY1^+^ FAPs have been associated with tissue-level fibrosis in the context of denervation (34), though not in Duchenne muscular dystrophy, in which an *Adam12*/*Mmp19*/*Postn*-expressing subpopulation (without a clear analog in our data set) appears to be a dominant contributor (53).

THBS1 promotes FAP proliferation and expansion of the overall FAP pool while particularly impacting the FAP2 subpopulation. Indeed, subtype profile and velocity plots of FAPs from HFD-fed KO mice did not demonstrate FAP2 enrichment and, indeed, were nearly indistinguishable from those of CD-fed WT mice. Dietary condition and *Thbs1* ablation had little impact on the relative size of the FAP1 subpopulation, an analog of previously described adipocyte progenitors (54). The observed FAP profiles, therefore, align with our previous findings: while DIO induces both increased intradiaphragmatic adiposity and fibrosis, FAPs isolated from the obese diaphragm do not display enhanced ex vivo adipogenesis but do exhibit upregulated ECM deposition. As such, increased intramuscular adipocyte number likely results from increased FAP number (27) or THBS1-dependent tissue remodeling creating a microenvironment permissive for adipocyte differentiation and expansion in vivo.

On the whole, THBS1 facilitates obesity-associated expansion of the diaphragm FAP pool, inducing a fibrogenic transcriptomic signature typified by TGF-β–dependent gene expression and enrichment of a THY1^+^ subpopulation previously linked to tissue-level remodeling. Given its multiple potential cells of origin, delineating the specific source of THBS1 responsible for the phenotype — and whether it reaches the diaphragm through circulation or from nearby adipose depots — is an important area of future investigation.

During a 6-month DIO time course, the diaphragm undergoes progressive anatomic remodeling characterized by increased intramuscular adiposity and ECM deposition (27). Here, we show that adipocytes in DIO mice are not uniformly distributed throughout the costal diaphragm tissue but instead exist largely in aggregations close to the rib attachment point. ECM distribution is also not homogenous, as densities of polymerized collagens and fibronectin often appear closely interposed with adipocytes. Similarly, THY1^+^ cells preferentially congregate near these intramuscular adipose depots, demonstrating a coupling of their presence and fibro-fatty expansion. *Thbs1* is an essential mediator of these processes, given that KO mice are protected from adipose depot expansion and the associated increase in THY1^+^ FAPs and ECM deposition.

Tissue-level transcriptomic analysis substantiates these histological findings, showing that, compared with the HFD-fed WT diaphragm, the HFD-fed KO diaphragm displays reduced expression of numerous stromal genes, particularly those associated with adipocytes and ECM-depositing FAPs (e.g., *Lep*, *Fn1*, *Fbn1*, *Prg4*, and *Mfap5*). Conversely, transcripts more highly expressed in the HFD-fed KO diaphragm include those specific to the myofiber (e.g., *Actn3* and *Pvalb*). This raises questions as to whether increases in myofiber-specific transcripts in the obese KO diaphragm are relative — i.e., occurring because there is less stromal tissue than in the obese WT diaphragm — or represent a protective effect of *Thbs1* ablation on myofiber preservation during overnutrition. Our histological data support the former possibility, as diaphragm thickness, myofiber size, and myofiber type are unchanged between diaphragms of HFD-fed WT and HFD-fed KO mice. Moreover, compared with diaphragms of CD-fed WT mice, the WT HFD diaphragm does not display pathological hallmarks of atrophy (centrally nucleated or angular myofibers) (27).

THY1^+^ FAPs are predominantly fibrogenic and contribute to the deposition of fibronectin and other ECM species near adipose depots (40, 54). Delineating other roles they might play in tissue-level remodeling — e.g., differentiation into adipocytes (55) or secondary promotion of adipose depot expansion through inhibition of myofiber maintenance — are important future directions.

Our current findings apply only to male mice. Subsequent studies including both male and female animals will be required to determine whether any aspects of obesity-associated diaphragm remodeling or FAP complement are sexually dimorphic (81) — a critical issue given recent reports of estradiol signaling to promote pro-fibrotic responses in *Pdgfra*-expressing mesenchymal cells of the abdominal wall musculature (82). Additionally, future application of a micronutrient-matched CD in parallel to standard chow can definitively rule out any impact of small vitamin and mineral concentration differences on the observed phenotypes.

Finally, while the single obese human diaphragm sample evaluated in this study contains large intramuscular adipose depots surrounded by fibronectin and THY1-immunopositive stromal cells, further analysis of human samples from individuals with normal and elevated BMI is required to determine the degree to which our animal model findings can be translated to humans—a key point given potential interspecies differences in intramuscular adiposity (83).

Our testing of isometric specific force in isolated diaphragm strips demonstrates that 6-month DIO markedly impairs contractile function. The process depends on *Thbs1*, as diaphragm samples from KO mice subjected to the same diet maintain equivalent specific force versus baseline and demonstrate significantly greater force than isolates from HFD-fed WT mice. In contrast, measurements of specific force in single myofibers isolated from HFD WT and HFD KO mice exhibit no difference between groups. This data can be interpreted as demonstrating that obesity-induced isometric force deficits result from tissue-level remodeling rather than myofiber dysfunction. We note that single fiber force testing must be performed on permeabilized myofiber segments. While fresh, intact myofibers can be isolated from small murine muscles like the lumbrical (84); the procedure is not technically feasible in larger muscles like the diaphragm (DRC, unpublished observations). Consequently, the single myofiber approach tests the functional integrity of the sarcomere but may exclude the assessment of extrinsic regulation of excitation-contraction coupling (85).

Despite these caveats, augmentation of intramuscular adipose depots likely plays a substantial role in diminution of diaphragm isometric force during overnutrition. For instance, in well-defined models of simultaneous intramuscular adiposity and contractile dysfunction (such as chemical injury of the extensor digitorum longus), lipodystrophic mice unable to generate adipocytes are protected from postinjury isometric force deficits (38). As we previously described in the obese diaphragm and again demonstrate here, simple occupation of muscle CSA by adipocytes was insufficient to quantitatively account for the measured isometric specific force reduction in WT mice (38). Together, these findings suggest that a negative impact of intramuscular adipose depots on contractile physiology may be exerted through disruption of normal tissue architecture or via paracrine signaling to myofibers (86). Clinical relevance is highlighted by reports linking reduced limb muscle density (computed tomography), indicative of elevated intramuscular adiposity, to reduced physical performance in elderly men (87) and impaired lung function in young adults with obesity (23).

In our study, *Thbs1*-dependent fibro-adipogenic remodeling and contractile dysfunction correspond with compromised diaphragm motion on noninvasive ultrasound, highlighting the manifestation of THBS1-driven changes in clinically measurable outcomes. Blockade of THBS1 has been shown to mitigate hyperglycemia-induced peritoneal fibrosis (88), while inhibition of THBS1-dependent TGF-β activation reduces renal injury and proteinuria in mouse models of diabetic nephropathy (89). To this end, pharmacological targeting of THBS1 and its downstream signaling pathways may hold potential as a treatment for obesity-associated respiratory dysfunction.

## Methods

### Sex as a biological variable.

Male mice were used for the study to obviate any confounding effect of variable estrogen levels on FAP biology (82). Diaphragm tissue from women of postmenopausal age revealed similar obesity-induced remodeling to that seen in male DIO mice.

### Animals.

WT C57BL/6J (strain 000664) and *Thbs1^–/–^* mice (strain 006141) were obtained from The Jackson Laboratory. Jackson maintains *Thbs1^–/–^* mice on a C57BL/6J background. Animals were housed in pathogen-free containment with a 12-hour light/12-hour dark cycle and ad libitum food and water. For DIO studies, mice received a normal chow diet (5L0D; LabDiet) until 2 months of age. CD-fed mice continued this for an additional 6 months, while HFD-fed mice switched to a diet containing 45% calories from lipid (D12451; Research Diets) and subsequently maintained this for 6 months. Body composition was assessed via NMR (using the EchoMRI 4in1-500). Glucose tolerance testing was performed via intraperitoneal injection of a 10% dextrose solution (in sterile water) dosed at 1 g/kg total body weight. Glucose measurements were made using a One Touch glucometer (Lifespan) on blood samples obtained from tail nicks at time points 15, 30, 60, 90, and 120 minutes after dextrose injection.

### Human tissue samples.

Diaphragm specimens from women were obtained from cadavers donated to the Anatomical Donation Program of the University of Michigan Medical School. Donors included 1 lean (BMI 22.0 kg/m^2^) individual (72 years old) and 1 individual with obesity (BMI 32.4 kg/m^2^) (62 years old) — neither with a medical history of congestive heart failure or primary pulmonary disease. Samples (approximately 4 × 4 cm) were obtained from the left costal diaphragm at the midpoint of the anterior-posterior axis, 2–3 cm medial to the rib attachment point. Samples were fixed for 48 hours in 4% paraformaldehyde at 4°C, then paraffin-embedded and sectioned (7μm thickness) in the transverse plane.

### Diaphragm ultrasonography.

Diaphragm ultrasonography was performed as previously described (71). Briefly, diaphragms were localized by ultrasound using a transversely oriented MS250 transducer (frequency 24 MHz) (Visual Sonics). Diaphragm motion, observed in M-mode, was recorded for 3 or more respiratory cycles. Excursion amplitude and inspiratory and expiratory velocities were measured on still images with values averaged over the recorded cycles.

### Ex vivo isometric force testing (muscle strips).

Isometric force testing on diaphragm strips was performed as previously described (27, 90). Briefly, tetanic force was measured on midcostal diaphragm muscle strips that were 2 to 4 mm wide. In a Krebs-Ringer bath containing 0.03 mmol/L tubocurarine chloride, held at 25°C and bubbled with 95% O_2_ and 5% CO_2_ (maintaining pH 7.4), an attached rib was sutured to a servomotor (model 305B; Aurora Scientific), and the free central tendon edge was sutured to a force transducer (model BG-50; Kulite Semiconductor Products). A field generated between 2 platinum electrodes by a biphasic current stimulator (model 701A; Aurora Scientific) was employed to electrically stimulate the bath. LabVIEW 2014 software (National Instruments) controlled the electrical pulse properties and servomotor activity while recording transducer data. Strips were adjusted to optimal length (Lo) — defined as the length at which a stimulus pulse elicited maximum isometric force (Po). Muscle CSA was calculated using Lo and muscle mass. Specific force was calculated as the quotient of Po/CSA.

### Ex vivo isometric force testing (single myofibers).

Single myofiber experiments were performed as previously described (91). Costal diaphragm fiber bundles (4 mm in length and 1 mm in diameter) containing longitudinal arrays of myofibers were manually excised from the central region of the muscle and then immediately immersed in ice-cold skinning solution — containing potassium propionate, imidazole, and EGTA (MilliporeSigma) — for 30 minutes before storage at –80°C in a solution containing 50% glycerol by volume. Prior to each experiment, bundles were removed from storage and thawed before the removal of individual fibers by manual extraction with fine forceps under a stereomicroscope. Researchers were blinded to the experimental group when performing this testing.

Force responses and motor position were acquired through a 16-bit A-D board (NI-6052; National Instruments) and analyzed on a computer running custom-designed LabVIEW software (National Instruments). The solution-changing system (model 802A; Aurora Scientific) consisted of 3 glass-bottom chambers housed in a moveable, temperature-controlled stainless-steel plate. Movement of the plate relative to the fiber was achieved via 2 stepper motors: one to lower and raise the chamber array and the other to translate the plate to a new chamber position.

Chamber 1 was filled with an EGTA-containing relaxing solution in which fibers could be manipulated. In this chamber, fibers were manually sutured to a servomotor (model 322; Aurora Scientific) force transducer (model 403A; Aurora Scientific) apparatus with USP 10-0 monofilament nylon suture. Optimal sarcomere length was defined based on the diffraction pattern of laser light passed through the mounted fiber (92). Once achieved, the corresponding optimal fiber length (Lf) was measured under a stereomicroscope. Fiber CSA was estimated (on fibers held at Lf) using width and depth measurements obtained from high-magnification digital images of top and side views of the fiber. Chambers 2 and 3, respectively, contained a low-[Ca^2+^] preactivating solution and a high-[Ca^2+^] activating solution. Fibers were exposed to Chamber 2 solution for a 3-minute priming period, during which the passive force required to maintain the fiber at Lf was measured. Fibers were then transferred to Chamber 3 to elicit maximum isometric force (Fo) during sustained contraction. Maximum total isometric force was calculated as the difference between Fo and passive force. Specific force was calculated as the quotient of maximum total isometric force/CSA.

### Single-cell isolation.

Diaphragmatic mononuclear cells were isolated through a protocol adapted from a previous study (28). Costal diaphragms were excised, minced with scissors, and digested in collagenase type II (Worthington Biochemical) diluted to 0.067% by weight in serum-free DMEM (Thermo Fisher Scientific). After a 1-hour incubation at 37°C, samples were triturated 3–4 times through an 18-gauge needle, then incubated at 37°C for an additional 10 minutes. Next, collagenase was inactivated with an excess of DMEM containing 10% fetal bovine serum (FBS); samples were sequentially passed through 100 and 40 μm cell strainers (Falcon, Corning) to remove debris. Erythrocyte lysis was achieved via 30-second exposure to hypotonic stress, after which cells were resuspended in phosphate-buffered saline (PBS).

Flow cytometry analysis used established marker profiles (28). Briefly, fresh cells were incubated in PBS with fluorophore-conjugated CD31, CD45, integrin α_7_, and Sca-1 antibodies at dilutions indicated in Supplemental Table 1 for 30 minutes at 4°C. DAPI was added for the final 5 minutes of the incubation to act as a dead cell marker. Cells were analyzed on a MoFlo Astrios EQ running Summit software (version 6.3; Beckman-Coulter). Gates were established using a fluorescence minus one approach, and plots were generated in FCS Express 7 (DeNovo Software).

### Cell culture.

After isolation, FAPs were cultured for 4 days in 12-well plates containing standard medium: DMEM with 10% FBS and antibiotic/antimycotic (penicillin, streptomycin, and amphotericin B from MilliporeSigma). Cells were then seeded at 30% confluence in optical bottom 96-well plates with standard medium and allowed to attach over 24 hours. The medium was then changed to DMEM with 1% FBS with/without THBS1 (5 μg/mL). After 3 days, cells were fixed, blocked, and immunostained as previously described (27) using antibody concentrations indicated in Supplemental Table 1. For indicated experiments, SB-413542 (MilliporeSigma), which impairs TGF-β receptor I– and II–dependent signaling (93), was added at 10 μg/mL, as previously described (66), in parallel with THBS1. For BrdU staining, cells were incubated in 10 μm BrdU (Abcam) for the 24 hours preceding analysis, then treated with 2 M HCl (20 minutes at room temperature, followed by 10 minutes at 37°C) to achieve DNA hydrolysis. For Ki67 staining, wash buffers and antibody diluents contained 0.2% Tween 20. Detergent was excluded for extracellular fibronectin staining. Counterstain with Alexa Fluor 488–conjugated phalloidin (Thermo Fisher Scientific) was used in specific experiments.

### scRNA-Seq and bioinformatics analysis.

All mononuclear cell samples for scRNA-Seq were isolated on the same day to obviate the need for batch effect correction. Sequencing was performed by the University of Michigan Advanced Genomics Core, with libraries constructed and subjected to 151 paired-end cycles on the NovaSeq 6000 platform (Illumina). Bcl2fastq2 Conversion Software (Illumina) was used to generate demultiplexed FASTQ files. Mapping and quantitation were also done by the Advanced Genomics Core, using the ENSEMBL GRCm38 reference and Cell Ranger to generate feature-barcode matrices and aggregate the results from different samples. For all samples, Q30 bases in unique molecular identifier (UMI) were >94%, reads mapped to the genome were >94%, and fraction reads in cell were >86%. Cell number in samples ranged from 3,774 to 4,369, while median UMI counts per cell ranged from 7,554 to 8,032. Approximately 10 million reads were sequenced per sample.

To produce velocity plots, velocyto (v. 0.17.17) and scVelo (v. 0.0.4, with Python 3.7.12) were used. Velocyto was installed as a conda environment. For each sample, the t-SNE coordinates were exported from the Loupe Browser with their barcode. Then velocyto was run from the command line with the t-SNE coordinates for each sample, a GTF file containing positions of repetitive elements to mask, the position-sorted BAM file of filtered raw reads, and the mouse GTF file “Mus musculus.GRCm38.98.gtf.” The output is a file in LOOM format, designed to efficiently store single-cell data sets and metadata. The LOOM files were input to an R (v. 4.1.3) script with the package reticulate (v. 1.25) loaded to run Python in R. The Python package scVelo was imported to the R script to calculate the cellular dynamics. For each sample, using the t-SNE coordinates and the calculated velocity, with ggplot2 (v 2.3.4) library loaded, a t-SNE plot could be produced with the rate and direction streams.

Using Loupe Browser 5, differential expression was calculated within FAP subclusters, comparing each sample versus the 2 others combined. This gave a table of *P* values and fold-changes that were uploaded to iPathwayGuide (https://advaitabio.com), using a linear absolute fold-change cutoff of 1.5. All 3 comparisons in a subcluster were combined as a meta-analysis in iPathwayGuide, to visualize Venn diagrams of the marker genes and corresponding pathways.

### Whole tissue gene expression profiling.

Costal hemidiaphragms were cleaned of adherent tissues, snap-frozen in liquid nitrogen, and digested in Trizol (Thermo Fisher Scientific) with mechanical homogenization. Total RNA was isolated with RNeasy reagents (QIAGEN) as per manufacturer’s protocol. Samples were subjected to quality control via measurement of RNA integrity number values (TapeStation analysis software v3.2, Agilent Technologies). QuantSeq 3′ mRNA sequencing (Lexogen) was performed by the University of Michigan Advanced Genomics Core. GSEA was performed using GSEA 4.1 software (University of California, San Diego). Volcano plots were generated in R Studio. For qPCR cDNA was synthesized with SuperScript II (Invitrogen) and the PCR performed with SYBR Green (Thermo Fisher Scientific) on a StepOnePlus machine (Applied Biosystems). Primer sequences for indicated genes (previously described) (27, 45) are as follows: *Lep* forward: CAGTGCCTATCCAGAAAGTC; reverse: ATCTTGGACAAACTCAGAATG. *Pdgfra* forward: TTGATGAAGGTGGAACTGCT; reverse: ATTCCTCTGCCTGACATTGAC. *Fn1* forward: CGTTCATCTCCACTTGAT; reverse: CAGTTGTGTGCTCCGATCTC. *Col3a1* forward: CTTCTGGTTCTCCTGGTC; reverse: CAACCTTCACCCTTATCTCC. *Emr1* forward: CTTTGGCTATGGGCTTCCAGTC; reverse: GCAAGGAGGACAGAGTTTATCGTG.

### Histological analysis.

Seven micrometer–thick formalin-fixed, paraffin-embedded sections of the costal hemidiaphragm were prepared as previously described (27) and included samples in both transverse and longitudinal planes with respect to myofiber orientation. Both sample types were approximately 2–4 mm wide and included tissue encompassing the entire rib to tendon extent of the costal diaphragm muscle. Longitudinal samples were analyzed along the entire rib-tendon length, while transverse sections were analyzed at the midpoint of the rib-tendon axis. H&E and Picrosirius red staining were performed by standard methods. Fiber size measurements were made using transverse sections stained with fluorescein 405-conjugated wheat germ agglutinin (Biotium) diluted 1:200 in HBSS and incubated for 30 minutes at room temperature. IHC for perilipin, THY1, and fibronectin was performed with primary-secondary antibody pairs as indicated in Supplemental Table 1. Staining of human tissue samples described above was performed using the same protocols and antibodies.

For myofiber typing analyses, excised costal diaphragm samples were sequentially submerged in 30% sucrose in PBS, then a mixture of 30% sucrose in PBS/OCT (1:1) until the tissues no longer floated. Tissues were subsequently placed in an OCT solution and frozen in liquid nitrogen–cooled isopentane for cryosectioning. Seven micrometer–thick transverse cryosections were immunostained with 2 primary antibodies specifying type I and type IIa fibers (type IIb and type IIx fibers were unstained) as previously described (38). Primary and secondary antibodies are indicated in Supplemental Table 1.

For immunofluorescence staining of CD68 and PDGFRα, excised diaphragm tissues were directly embedded in OCT and quickly snap-frozen in liquid nitrogen-cooled isopentane. Seven micrometer–thick transverse or longitudinal cryosections (as described above) were fixed in 4% paraformaldehyde/PBS for 5 minutes at room temperature, then blocked and permeabilized in 1% BSA/PBS or Mouse on Mouse (M.O.M., Vector Laboratories) blocking medium containing 0.5% Triton X-100. Tissue slides were stained with different combinations of primary antibodies in 1% BSA/PBS or MOM antibody medium containing 0.1% Triton X-100 overnight at 4°C, followed by corresponding secondary antibodies for 1 hour at room temperature. Primary-secondary antibody pairs are indicated in Supplemental Table 1. Nuclei were counterstained with DAPI (diluted in deionized water) for 5 minutes at room temperature before mounting of samples with ProLong Diamond (Invitrogen).

Samples were imaged using an Olympus DP72 camera mounted on an Olympus SZ61 microscope or a Nikon A1 confocal microscope running NIS-Elements software (Nikon). For all forms of staining, at least 3 sections, separated from one another by at least 100 μm, were analyzed by individuals masked to the experimental group, and quantitative morphometry was performed using NIH ImageJ.

### Statistics.

Statistical analysis was performed in GraphPad Prism 10 and employed Student’s 2-tailed *t* test for 2-group comparisons, 1-way ANOVA (with Tukey’s post hoc test) or Kruskal-Wallis nonparametric test for comparisons of 3 or more groups, 2-way ANOVA (with Holm-Šídák post hoc test) for 2 variables, and linear regression for correlation analysis. *P* < 0.05 indicated statistical significance. Quantitative data are shown as mean ± SD.

### Study approval.

The University of Michigan Institutional Animal Care and Use Committee approved all animal studies.

### Data availability.

Sequencing data were made publicly available through upload to National Center for Biotechnology Gene Expression Omnibus (GSE241005). Accession numbers are GSM7713701 (WT CD), GSM7713702 (WT HFD), and GSM7713703 (KO HFD). All other raw data values are provided in the Supporting Data Values file.

## Author contributions

EDB and THC conceived of the study. EDB and THC designed the experiments with advice from DRC, and SVB, EDB, MSW, RKV, SHK, CSD, and KCB performed the experiments. EDB and THC analyzed the data with advice from SVB. EDB and THC wrote the manuscript, and DEM and SVB edited the manuscript.

## Supplementary Material

Supplemental data

Supporting data values

## Version 1 07/02/2024

In-Press Preview

## Version 2 08/22/2024

Electronic publication
